# Major Choices: Students’ Personal Intelligence, Considerations When Choosing a Major, and Academic Success

**DOI:** 10.3390/jintelligence12110115

**Published:** 2024-11-13

**Authors:** Kateryna Sylaska, John D. Mayer

**Affiliations:** 1 Department of Psychology, University of New Hampshire, Durham, NH 03824, USA; 2 Department of Psychological Science, Carthage College, Kenosha, WI 53140, USA; ksylaska@carthage.edu

**Keywords:** personal intelligence, personality, college major, major choice, academic success

## Abstract

A student’s choice of major is influenced by their parents and peers, as well as by the quality of the college department that offers the major and by broader cultural and economic issues. The student’s own personality, including their ability to reason about themselves and their interests, also contributes to the choice and its outcomes. In a preliminary study, we developed a Choice of Major Scale that depicts key aspects of students’ consideration of their major. Then, across three studies (Ns = 304, 466, and 492), we examined the students’ personal intelligence, defined as their capacity to reason about their own and other people’s personalities, as well as a Choice of Major Scale, and the relation of those variables to important academic outcomes. The results depicted a pattern that the students who better understood personality and were more engaged in choosing a major, compared to others, considered more majors initially, chose a major more quickly, and exhibited better academic outcomes subsequently.

## 1. Introduction

A student chooses a college major in the context of contemporary societal pressures; for example, societal encouragement to major in the STEM disciplines in the 2000s and economic conditions that favor one field of study over another (e.g., Bybee 2013). Parents often have a say about the choice of major, as do students’ friends (Ryan and Deci 2000) and influential professors. But surely, part of the decision involves students’ own understanding of their interests and motives and how those relate to their choices. The choice of major is a matter of some consequence for both the student and for the colleges and universities they attend. A poorly chosen major can delay a student’s progress or lead to dissatisfaction with their institution (Allen and Robbins 2010; Schmitt et al. 2008; Wessel et al. 2008).

Here, our focus is on college students’ psychological abilities and related considerations when choosing a major area of study. There are, of course, many reasons why students may choose a major that suits them—or fail to. We examine whether some students may be more skilled in making the choice than others. Understanding such differences among students may lead to new ways to assist their decision process—a complex balancing act between the student’s own interests and talents and their social and economic needs (Wiswall and Zafar 2021). Understanding this process as an intellectual pursuit has real-world policy implications for educational institutions, such as when to require students to declare a major (Spight 2020).

We ask whether students higher in personal intelligence—the ability to understand personality in themselves and other people—make better choices regarding their major. For example, such students may better understand the personal attributes they need to ‘fit’ to their major and, later, their careers (e.g., Allen and Robbins 2010). If that were so, it could lead to (a) better understanding of how students choose the right major, (b) better support for students to understand their own personalities, and (c) better student retention on the part of institutions (Allen and Robbins 2010; Schmitt et al. 2008; Wessel et al. 2008).

### 1.1. Personal Intelligence 

Personal intelligence (PI) can be assessed through ability-based measures of accuracy in understanding personality. The term *personal intelligence* was chosen to parallel the terms emotional intelligence and social intelligence (Mayer 2014), and like those two broad areas of intelligence, personal intelligence (PI) involves people-centered reasoning and correlates with other ability measures in the area (Bryan and Mayer 2021). Personal intelligence can be distinguished conceptually from emotional intelligence in that reasoning about personality is broader than reasoning solely about emotions and includes reasoning about motives, personal constructs, and self-control. Students that are higher versus lower in PI exhibit distinct patterns in their college lives: they manifest closer relationships with their peers, are more conscientious in their studies, and are less likely to engage in agonistic, problematic behavior on campus (Mayer et al. 2024). The specific focus here is whether those students also use their ability to align their personality to their college major. Although we focus on college, this process may be a harbinger of their later choice of career (e.g., Holland 1966; Ishitani 2010).

To explore such issues, we introduce the Choice of Major Scale, a new measure that asks students, in relation to their majors, about their level of motivation, their desire to make the choice themselves, and their satisfaction with the choice overall. We then examine personal intelligence and the Choice of Major Scale in relation to students’ academic processes and outcomes including, for example, the number of alternative majors they considered, their class attendance, and their GPA.

The assessment of personal intelligence can be carried out with scales such as the Test of Personal Intelligence (TOPI, Mayer et al. 2019). The TOPI, which is implemented across parallel test forms of different lengths, employs multiple-choice questions with correct or incorrect answers keyed to the research literature in personality psychology. For example, one item reads:

A person is straightforward and modest. Most likely, she could also be described as:

(a) Valuing ideas and beliefs;

(b) Active and full of energy;

(c) Sympathetic to others and tender-minded;

(d) Self-conscious and more anxious than average.

The correct answer—“sympathetic to others and ‘tender minded”—is in this instance keyed to findings by Ashton and Lee (2009).

One can think of intelligences, broadly conceived, as concerned chiefly with either reasoning about things, reasoning about people, or some combination of the two. Although PI’s focus is on reasoning about personality generally (rather than emotions exclusively), PI correlates with other people-centered intelligences, for example, *r ≈* 0.70 with emotional intelligence assessed as an ability, *r* = 0.40 with general comprehension (of both people and things), and less highly, at *r* ≈ 0.20 with the more thing-centered visuo-spatial intelligence (e.g., Bryan and Mayer 2021). PI correlates with expert-scored cultural intelligence responses *r* = 0.28 and with self-compassion (Mowlaie et al. 2017; Sternberg et al. 2022). PI is associated with the ability to accurately describe others’ personalities in both young adults and children (Allen and Mayer 2022). People higher in PI also exhibit more constructive relationships at school, exhibit less interpersonal conflict on lifespace (biodata) measures, and attend more conscientiously to classwork (Mayer et al. 2024).

It is not just enough to have the *capacity* to reason in the area, however. One also must be willing to engage in reasoning about a major. For that reason, we also examine students’ reports of their inner experiences of their academic pursuits, using the *Choice of Major Scale* (CMS) developed here for that purpose. The CMS examines three areas: (a) students’ satisfaction with their major, (b) motivation, and (c) sense of alignment with classmates with the same major.

Beginning in the 1960s, John Holland described occupations as having “personalities” (e.g., Holland 1966; McDaniel and Snell 1999). The same might be said of college majors: artistic, creative college majors draw students who are a bit unconventional; majors related to accounting and medicine draw students who are conscientious and responsible; and entrepreneurial fields draw those who enjoy interactions and networking with others, among other occupational types. Research using the Big Five personality traits (e.g., Goldberg 1993; John and Srivastava 1999), as another example, indicated that social science majors were more extraverted and higher in conscientiousness than English and fine arts majors; the latter are higher in neuroticism by comparison (e.g., De Fruyt and Mervielde 1996; Larson et al. 2010). Psychology majors are high in social interests but neither particularly artistic nor conventional (De Fruyt and Mervielde 1996; Harton and Lyons 2003; Pringle et al. 2010). It seems at least possible that college students higher in PI will exhibit more successful engagement in choosing a major than will students lower in PI.

### 1.2. Overview of the Present Research Studies

We were interested in both students’ objectively measured PI, as well as their subjective considerations of their choice of major, and the relation of both to success in choosing a major. To capture students’ subjective experience, we developed a Choice of Major Scale (CMS) specifically for the current studies, using data from the three studies reported here, and report it in its final form first. This is followed by Studies 1, 2, and 3, which examine students’ TOPI and CMS scores in relation to real-life indices of college student performance captured by the Academic Major Criterion Items (AMCI). The AMCI consists of lifespace items that are objective and potentially verifiable (Asher 1972; Furnham 2017; Mayer and Bryan 2024) and relevant to criteria of college student success as identified in earlier research. In its final form, the AMCI consisted of 14 lifespace items that inquired as to the students’ grades in their major, hours studied, course attendance, decision making related to their major, and educational satisfaction, among other qualities.

Study 3 extends the research by assessing personality–major fit using independent norms of the ‘personality’ of majors supplied by the research arm of the ACT organization. These studies were based on the first author’s doctoral thesis (Sylaska 2016).

## 2. Choice of Major Scale (CMS): Development and Factor Structure

To assess students’ subjective reports of their process for selecting and choosing a college major, we drew on data from a preliminary pool of 79 items employed across the three studies here and, from those, developed a 26-item Choice of Major Scale (CMS) to represent students’ viewpoints.

### 2.1. The Provisional Scale

After reviewing relevant literature, the first author identified 11 content areas important to how students choose their majors (e.g., Downey 2011; Malgwi et al. 2005; Soria and Stebleton 2013). She then interviewed 21 students and recorded their reflections about their choice of college major. Their relevant comments were compiled in a list classified according to the 11 areas. Test items were written to reflect their comments (e.g., “This major is very prestigious”) and also to reflect observations of similar prior research (e.g., Beggs et al. 2008; Downey 2011). From this inclusive list, we combined similarly phrased items until we arrived at an initial 79-item scale.

The 11 areas of items were then consolidated into three broader groups: (a) *satisfaction with Major*, which combined the two areas (of eleven) of confidence and commitment and focus; (b) *motivation,* which combined four areas: valuing the work, autonomous effort, autonomy of choice, and external rewards; and (c) *thinking about the major,* which combined five areas: choice exploration, personal growth, fit with other plans, match with other students in the major, and match to oneself. Individual items were answered on a four-point Likert scale anchored by strongly disagree, disagree, agree, and strongly agree.

The final version of the Choice of Major Scale was based on factor analyses of the three areas and consisted of an overall “Positive Choice” scale and six subsidiary scales. These analyses are described next.

### 2.2. Samples and Screening

The three data sets all used the complete 79-item measure. The data included a student sample from the college of liberal arts at a large public university in the Northeastern US (Study 1, *N* = 304), self-identified college students from MTurk (Study 2, *N =* 466), and a further sample of undergraduates (Study 3, *N* = 492) enrolled in psychology courses at the same university as Study 1. Briefly, the Study 1 and 3 samples were predominantly White, between 18 and 21 years of age, and included more women than men. The Study 2 sample from MTurk in 2016 was more diverse in ethnicity and age and more evenly divided between women and men. Participants from all three studies were screened, and a small number were removed using criteria for (a) excess missing data, (b) long-string responding, (c) “flatlining”, and (d) speedy completion. Details of the three samples are described in the individual studies that follow.

### 2.3. Analyses and Results

#### 2.3.1. Creating a Factor Model of the CMS

We sought to create a conceptually meaningful, replicable, simple-structure factor analytic model of the CMS—one in which each test item was loaded on just one scale. Because factor analysis is a large-sample technique, the model of the scale was constructed using data from Studies 1 and 2 combined and cross-validated on data from Study 3. Participant responses to the CMS were treated as categorical given its 4-point scale. The EFAs employed a weighted least squares mean and variance adjusted estimation (WLSMV) in Mplus, followed by an oblique geomin rotation, which optimized the distinction between factors. We sought to meet criteria of root mean square error of approximation (RMSEA) of <0.06, and Tucker–Lewis and comparative fit indices (TLI and CFI) near or >0.95 (Hu and Bentler 1999). Details can be found in the Technical Supplement (Mayer and Sylaska 2024, Part 2).

#### 2.3.2. Exploratory Factor Analyses

Applying EFAs to the three scale areas individually, the *Satisfaction* area items divided into Self-Confidence and Difficulty factors; the *Motivation* area into Intrinsic Motivation and External Rewards; and the *Thinking about Major* area into Decision Avoidance and Personality Alignment. We also fit an EFA to the 79 items in their entirety, but that yielded a considerably messier solution with a poorer fit (see the Technical Supplement, Mayer and Sylaska 2024, Appendix Supp. A, for details).

#### 2.3.3. Confirmatory Factor Analyses

We next constrained the analyses to fit a simple structure factor model to each of the three areas. To accomplish this, we selected a subset of items that loaded approximately λ > 0.60 on their primary factor, with secondary loadings λ < 0.20 on any other factor. For the *Satisfaction*, *Motivation*, and *Thinking* areas, this led to groups of 9, 11, and 13 items. By proceeding this way and dropping several additional items due to poor fit, we were able to model two-factor CFAs of each area, indicated in the top rows of Table 1.

**Figure 1:**
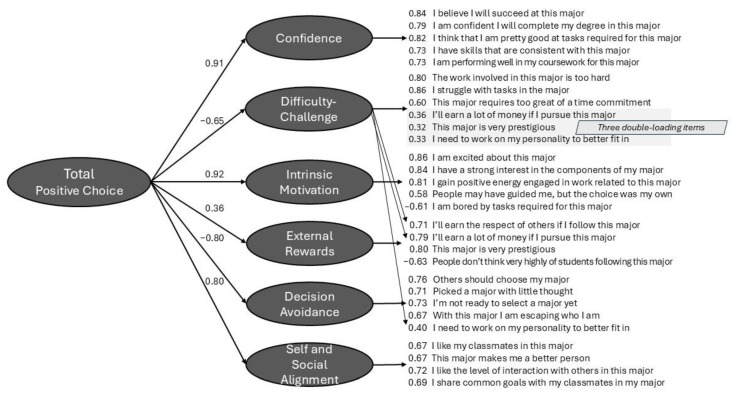
A confirmatory simple-type structure factor model of the Choice of Major Scale. The solution yielded one overall “Positive Choice” factor and six more specific factors from Confidence to Self and Social Alignment. Items in the diagram are sometimes abbreviated. Each item loads on one factor with the exception of three items from Difficulty-Challenge, of which two had primary loadings on External Rewards and one on Decision Avoidance.

We next tested a hierarchical model to combine the earlier scales—one that included an overall factor that we labeled Positive Major choice, and nested within it the individual six factors and their 26 items (the three conceptual areas were not included as they would have just two indicator factors each). The positive orientation of the Major Choice factor reflected that four of the six subscales indicated a positive sense of the major, including for example, Confidence and Intrinsic Motivation (see Figure 1), whereas two of the six subscales (i.e., Difficulty-Challenge and Decision Avoidance) reflected a more negative sense of the major choice process. We tested the hierarchical model both as-is and allowing three items that reflected a student’s difficulty with the major to double load. The fit statistics were good for both solutions. A diagram of the model including final 26 items and their loadings estimated for the combined Study 1 and 2 samples (*N* = 770) appears in Figure 1.

We then cross-validated the models on the Study 3 sample. For the most part, the fits were similar and just slightly lower (Table 1, bottom rows). We did, however, drop 1 further item to arrive at the final 26 to improve the fit in the Study 3 sample.

#### 2.3.4. Factor-Based Scales and Scale Reliabilities

Factor-based scales were constructed by taking the participant’s average response to the given scale’s items. The total score did the same for all 26 items. The coefficient alpha values of the CMS scales across the three studies ranged from: Overall, *α* = 0.73 to 0.75; Confidence, 0.76 to 0.84; Difficulty, 0.53 to 0.74; Intrinsic Motivation, 0.67 to 0.71; External Rewards, 0.57 to 0.65; Decision Avoidance, 0.65 to 0.79; and Social Alignment, 0.67 to 0.72. The CMS scale and scoring instructions can be found in the Technical Supplement (Mayer and Sylaska 2024, Appendix Supp. B).

### 2.4. Discussion of the CMS Measurement Study

The CMS represents at least some of the main concerns students express using a seven-scale approach based on the overall factor and six sub-factors. The overall factor represented the student’s positive feelings toward their choice; the six more specific scales reflected (a) Confidence, (b) Difficulty-Challenge (c) Intrinsic Motivation, (d) External Rewards, (e) Decision Avoidance, and (f) Self and Social Alignment. The model fit well but could be improved by allowing three items—two items primarily reflecting external rewards (prestige and monetary gains), and one primarily reflecting Decision Avoidance (e.g., need to “work on my personality to fit in”)—to also load on the Difficulty-Challenge factor. Conceptually, students who chose majors they found personally difficult often did so with an eye to the social and monetary rewards that might ensue. The CMS provides a reflection of students’ internal experience, which is useful for understanding their actual academic performance and other outcomes. The final scale’s correlations with criteria are reported across the next studies. The scale is available in Appendix Supp. B of the Technical Supplement (Mayer and Sylaska 2024).

## 3. Study 1: Choice of Major and Academic Outcomes

In Study 1, participants were asked to complete a survey containing the TOPI, CMS, and a number of questions concerning their choice of major and academic performance with the purpose of examining two hypotheses: First, we hypothesized that higher personal intelligence would correlate with higher levels of positive choice on the CMS regarding one’s major. Second, we hypothesized that higher personal intelligence and CMS positive choice both would relate to definable academic outcomes as measured by the Academic Major Criterion Items (AMCI).

### 3.1. Study 1 Measures and Method

#### 3.1.1. Study 1 Recruitment, Screening, and Final Sample Characteristics

Participants were recruited from a state university’s college of liberal arts in cooperation with the dean’s office of the college. The dean’s office issued an e-mail at mid-semester inviting students to participate in a brief online survey about their major. Students were told their participation would help the institution better understand the process of choosing a major and were further incentivized by being entered in a raffle to win one of four USD 25 gift cards for their participation. The 2017 enrollment in the college can be estimated at roughly 3800 students based on the incoming class of 1031 (more detail is in Mayer and Sylaska 2024) and estimated retention rate of 87%. The 482 logins to the survey therefore represented an approximate response rate of 12–13%, although not all of those who logged in completed the survey.

Screening protocol: Of the 482 participants who logged onto the survey, 178 participants were flagged for either missing data on 25% or more of the survey, answering questions in 2 s or less per item (Curran 2016; Huang et al. 2012) or both. No participants who completed the survey exhibited repetitive responding on the overall CMS or its last 25 items, defined as key-pressing 67% or more of the same response alternative. The flagged participants were removed, leaving *N* = 304 participants included in the Study 1 analyses.

Demographic characteristics: Of the 304 participants of Study 1, 230 (76%) identified as women and 74 (24%) as men. Nearly all (*N* = 299) were between the ages of 18 and 23; three were 25 years old or older. The group was predominantly White (92%), with some Asian (5%) and Multiracial (2%) persons being represented. The most common majors were psychology, undeclared, and communication.

#### 3.1.2. Study 1 Materials

Test of Personal Intelligence MINI-12 (TOPI MINI-12): The Test of Personal Intelligence—MINI (TOPI MINI) is an ability-based, 12-item IRT-informed scale of personal intelligence that correlates in the *r* = 0.80s with longer versions of the scale. All TOPI items employ a multiple-choice format with one alternative credited as correct (“1-point”) and three distractors earning no credit. See the Introduction to this paper for a sample item.

Choice of Major Scale (CMS): The CMS consisted of the 26 items described in the earlier “Choice of Major Scale…” section.

Academic Major Criterion Items (AMCI): The *Academic Major Criterion Items* were 14 items yielding 12 variables indicating (a) the number of alternative college majors the student had considered, (b) their currently considered options, (c) the number of semesters taken to decide on a major, (d) grades in the major and overall, (e) time spent studying, (f) course absences, and (g) desire to withdraw from college. Also included was the number of courses taken. The students’ GPA was asked about in two ways: to list their GPA and “the most common” grade they received. If students did not know their GPA, “the most common grade” was converted to a GPA and substituted for it (this affected about 2% of the responses). The AMCI items are best considered lifespace data: criteria that are objective and potentially verifiable (Asher 1972; Furnham 2017). For example, Kuncel et al. (2005) found that self-reported GPA strongly correlated with GPA identified on students’ transcripts, *r* = 0.82. More generally, lifespace items such as these—also called biodata (i.e., ‘biographical …’)—are an important source of data in the field (e.g., Furnham 2017; Mayer and Bryan 2024; Mount et al. 2000; Stokes 1999).

### 3.2. Study 1 Results

#### 3.2.1. Study 1 Descriptive Statistics

Table 2 contains the descriptive statistics for the central variables of Study 1. To the far left are the variable names, followed by the possible range for each, the mean, and SD. Most values were as-expected except that Study 1 exhibited a restriction of the range: the mean proportion correct for the TOPI was higher than usual at *M* = 0.87 versus *Ms* ≈ 0.76 in Studies 2 and 3. Given the participant’s generally high performance, the variance for Study 1 v. Studies 2 and 3 was commensurately lower at *S*^2^ *=* 0.39 v ≈ 0.50, or 22% less variance in Study 1. This suggested that the Study 1 findings relevant to the TOPI might be weaker than those obtained in Studies 2 and 3.

#### 3.2.2. Study 1 Tests of Hypotheses

Did higher personal intelligence correlate with higher levels of positive choice regarding one’s major? Table 3 shows the correlations between the TOPI MINI and the scores of the CMS across studies. Contrary to expectation, there was no relation between the two overall scores, *r* = 0.03, *n.s.*, in Study 1. The one relation evident was that students with higher TOPI scores reported lower Difficulty-Challenge regarding their major, *r* = −0.23, *p* < 0.001. The first hypothesis was, at best, partially supported.

Did the TOPI and the Choice of Major Scale Total correlate with better academic outcomes? Table 4 indicates the Study 1 relations between TOPI, CMS, and GPA with the academic outcomes. Generally, both the TOPI and SCM results correlated with the students’ overall (self-reported) GPA, *r* = 0.21 and 0.16, respectively, *p*s < 0.001. There was a hint that students with higher TOPI scores achieved their GPAs with fewer hours of study at *r* = −0.10, *p* < 0.08 for the in-major hours in Study 1. Students with higher GPAs also exhibited fewer class absences overall at *r* = −0.32 in major and −0.22 for non-major courses, *p*s < 0.001.

## 4. Study 2: The MTurk Replication

Study 2 was a replication of the basic correlational design carried out in Study 1 but relied on a more diverse national sample of students recruited via Amazon’s Mechanical Turk in 2017. The earlier two hypotheses were carried forward from Study 1: that (a) students’ TOPI scores would be positively correlated with their positive processes around choosing a major and (b) both the TOPI and CMS scales would relate to academic criteria.

### 4.1. Study 2 Measures and Method

#### 4.1.1. Study 2 Recruitment, Screening, and Final Sample Characteristics

Recruitment of the second sample on Mechanical Turk (MTurk) began in 2017, soliciting adults aged 18 or older currently attending a college or university in the United States. In 2017, MTurk survey respondents produced relatively valid protocols after standard screening practices were applied. The payment rate of USD 0.50 for a survey of varied lengths including 30 min. was consistent with research recommendations at the time (e.g., Buhrmeister et al. 2011).

Screening protocol: The same screening protocol was employed as for the COLA study, i.e., for missing data, repetitive responding, speeding, or flatlining, the latter on the CMS. In addition, participants needed to correctly answer at least five or more of eight newly added attention check items. In all, 66 participants were flagged for missing data, 74 for speeding, 21 for passing fewer than 6 of the 8 attention checks, and none for flatlining. Any case with one or more flags was removed, leaving a final sample of *N* = 466.

Demographic characteristics: Of the 466 participants of Study 2, 238 (51%) were women and 225 (48%) were men. Of these, 263 were between the ages of 18 and 24, and 203 (44%) were 25 years old or older. The 466 participants were predominantly White (70%), with representations of Black (11%), Asian (7%), Hispanic (6%), and other groups. The three most common majors were Business Administration, Computer Science, and Psychology.

#### 4.1.2. Study 2 Measures

Measures were the same as in Study 1 with the exception of the addition of the aforementioned attention check items.

### 4.2. Study 2 Results

#### 4.2.1. Study 2 Descriptive Statistics

The MTurk group exhibited noticeably more variability on the TOPI relative to the Study 1 sample. Other than that, most variable *M*s and *SD*s appeared stable across studies. The descriptive statistics are in the second set of columns in Table 1.

#### 4.2.2. Study 2 Tests of Hypotheses

Did higher personal intelligence correlate with higher levels of positive choice regarding one’s major? The correlations between the TOPI MINI and the CMS scores are in Table 2 in the middle rows. In Study 2, the first hypothesis was upheld, with the TOPI and CMS Positive Choice relation at *r* = 0.33, *p* < 0.001. Consistent with that finding, the TOPI correlated positively with CMS subscales such as Confidence and Intrinsic Motivation at *r*s = 0.31 and 0.21, *p*s < 0.001, and negatively with the negative choice subscales of Difficulty and Decision Avoidance, *r*s = −0.32 and −0.46, *p*s < 0.001.

Did the TOPI and the Choice of Major Scale Total correlate with better academic outcomes? Table 3 indicates Study 2’s relations between TOPI, GPA, and CMS with the academic outcomes. As in Study 1, the TOPI and CMS correlated with the students’ overall reports of their GPA, *r* = 0.19 and 0.23, *p*s < 0.001 (see Table 4). There was again an indication that higher-scoring TOPI students needed to study less than other students to achieve their grades, *r* = −0.19, *p* < 0.001, for non-major courses but not for major courses. The TOPI also correlated with number of majors considered, *r* = 0.12, and fewer changes in major, *r* = −0.16, *p*s < 0.01 and 0.001. Students with higher TOPI and CMS scores and better GPAs appeared to declare their majors more quickly than others, although only GPA reached statistical significance: *r*s = −0.07, −0.08 and −0.10, n.s., n.s., *p* < 0.05. For this study, there was no relation between absenteeism and TOPI, GPA, or CMS.

### 4.3. Study 2 Discussion

Some key results from Study 2 were that PI, assessed by the TOPI, correlated substantially with CMS Positive Choice, indicating that students higher in PI exhibited more confidence, intrinsic motivation, less decision avoidance, and found it less difficult to complete work related to their majors compared with others. The relations were more robust than in Study 1 where, although most relations were in the same direction, only the lower Difficulty level was significant.

As in Study 1, the TOPI was positively related to GPA, and the trend toward needing less study time evident in Study 1 reached significance in Study 2. In addition, the TOPI was related to considering more alternative majors when beginning college, accompanied thereafter by fewer changes in major once a major was declared.

Some of the differences in findings were likely due to the differences between the Study 1 and Study 2 samples. The Study 1 sample consisted of traditional college students who volunteered to respond to the survey in response to a request from the Dean’s office; the Study 2 sample included more nontraditional students who were participating as part of their online activities and received a small payment. The participants in Study 1 were noticeably higher performing on the TOPI scale and exhibited 22% less variance than those in Study 2 (see “Descriptive Statistics” under Study 1); the restricted range likely reduced some of the correlations. A further study was conducted to better identify the pattern of results and to extend them.

## 5. Study 3: Replication and Extension with Psychology Majors

Study 3 was a further replication of the basic correlational design carried out in Studies 1 and 2, but with several additional measures to extend the research. These included a short measure of the HEXACO (Big Six) personality traits, student-reported SAT and ACT scores, and an enlargement of the academic criteria to include planning and seeking academic advising. Also added were scales of school burnout, academic adaptability, career interests, and an assessment of whether students with higher TOPI and Choice of Major Scale scores better matched their interests to their major (see Section 5.1).

Student reports of GPAs and SATs can be regarded as lifespace indices in that they are in principle verifiable, and students’ reports in this area tend to be quite accurate although not perfect. For example, the correlation between students’ reports of their GPAs and the GPAs on their transcripts is high at *r* = 0.85 (Sticca et al. 2017), as is the correlation between their reports of SAT scores and actual SAT scores at *r* = 0.89 (Cole and Gonyea 2010). Further, the SAT is a reasonable proxy for general intelligence (*g*) as indexed by IQ given the SAT’s correlation of *r* = 0.73 to 0.82 with IQ measures (e.g., Coyle 2015; Frey 2019; Frey and Detterman 2004). We capitalized on those relations by using students’ reports of their SATs as an approximate control for *g* in some of the analyses reported in Study 3.

The earlier two hypotheses were again tested: (a) students’ personal intelligence would be positively correlated with their positive considerations around choosing a major and (b) both the TOPI and CMS would relate to academic criteria. The latter hypothesis was augmented by adding new criteria to the AMCI around academic advising and the match between one’s interests and choice of major. A new hypothesis added here was that (c) the HEXACO would be generally unrelated to the AMCI variables.

### 5.1. Study 3 Measures and Method

#### 5.1.1. Study 3 Recruitment, Screening, and Final Sample Characteristics

Participants were recruited from the psychology department of the same university as Study 1. The study was one of several that students could select from on the Sona Systems platform, for which they received course credit in exchange.

Screening protocol: Overall, 536 participants logged onto the study. The same screening protocol was employed as in Studies 1 and 2. Of the 536, 42 were flagged for missing data, 20 for speeding, and none for repetitive responding or flatlining. Removing 44 flagged participants (some exhibited both issues) left a final sample of *N* = 492.

Demographic characteristics: Of the 492 participants of Study 3, 335 (68%) were women and 154 (31%) were men. Of these, 487 were between the ages of 18 and 24, and 5 were 25 years old or older. The 492 participants were predominantly White (92%), with representations of Hispanic (6%), Asian (3%), Black (2%), and Multiracial (2%) groups and a few others. The three most common majors were psychology, undeclared, and biomedical sciences.

#### 5.1.2. Measures

Test of Personal Intelligence-4G-45: The Test of Personal Intelligence-4G-45 was a 45-item subset of the TOPI 4; (Mayer et al. 2019); it exhibited a reliability of *α* = 0.91 in this sample.

Choice of Major Scale (CMS): The CMS consisted of the 26 items described in Section 2.3.3 and Section 2.3.4.

Academic Major Criterion Items (AMCI) (Augmented): The AMCI was repeated from Studies 1 and 2 with new items added concerning academic advising. “Pre-advising Preparation” included a count of planning behaviors the student engaged in before meeting with their advisor (e.g., “Make a list of a few courses I am interested in”). The “Expanded Advising” item was a count of more general advising opportunities accessed, such as taking vocational interest scales or attending walk-in workshops conducted by the university. Note that these two AMCI advising items were distinct from the ‘Academic Advising Experience Scale’ described next.

Academic Advising Experience Scale: The Advising Experience Scale consisted of 14 items answered on a 5-point Likert-Scale: *strongly disagree, disagree*, *neither agree nor disagree*, *agree, strongly agree*, and drawing on content relevant to evaluating advising (Winston and Sandor 2002a, 2002b). A principal axis EFA of the Advising Experience Scale indicated that the first unrotated factor loaded 11 of the 12 items > |0.25|. It represented negative advising experiences. We summed five items loading at or above *r* = |0.40| to create a factor-based scale to represent such experiences. We note that a higher dimensional solution might also be possible, but it might be advantageous in that case to add items over and above those we used, for example, the second unrotated factor loaded just two items > |0.30|.

Match to interest: The Match to Interest index is the correlation between the student’s work interests and corresponding profile of their chosen major. The students’ interests were measured by UNIACT-S, a 72-item measure assessing individuals’ occupational interests in the six Holland areas: Realistic, Investigative, Artistic, Social, Enterprising, and Conventional, with scale reliabilities that range from α = 0.83 to 0.89. Scores were converted to a T-score based on ACT norming samples (ACT 2009). The students’ choice of major was coded using the National Center for Education Statistics (NCES n.d.; NCES 2013), and the deidentified information was shared with the ACT organization. Using a proprietary system, they then returned a correlation between each student’s six scores on the UNIACT and the average interest profile of students in that major calculated on the same six scales of an independent sample. The congruence correlation exhibits a reported test-retest reliability of *r* = 0.76 (Allen and Robbins 2010; Arthur et al. 2006).

Academic Adaptability (AA): The Academic Adaptability scale is a nine-item adaptability scale drawn from the I-ADAPT (Wessel et al. 2008) reflecting students’ capacity to adapt to a new environment both in terms of learning and adjusting to uncertainty. A sample item is “I quickly learn new methods to solve problems” and is answered on the same 5-point Likert scale as the Academic Advising Experience Scale described above.

School Burnout: The School Burnout Inventory (Salmela-Aro et al. 2009) addresses (a) exhaustion at school (e.g., I feel overwhelmed by my schoolwork), (b) cynicism toward the meaning of school (e.g., I feel a lack of motivation in my schoolwork and often think of giving up), and (c) a sense of inadequacy at school (e.g., I used to have higher expectations of my schoolwork than I do now). Students indicated their level of agreement on a six-point Likert-type scale ranging from 1 (Completely disagree) to 6 (Completely agree). The overall scale score was calculated as the mean endorsement across items. Higher levels of school burnout are associated with lower levels of academic achievement and school engagement (Salmela-Aro et al. 2009). The Cronbach α for this study was 0.89.

Brief HEXACO Inventory. The Brief HEXACO Inventory is a 24-item version of the ‘Big Six’ personality inventory (BHI, de Vries 2013), designed to enhance validity in part by accepting lower reliabilities of a briefer scale (see Burisch 1984; Credé et al. 2012, for the rationale). The scale typically exhibits reliabilities ranging from *α* = 0.44 to 0.72 for the six scales (see de Vries 2013, Table 1); here, they ranged from *α* = 0.40 to 0.60. These alphas place upper limits on the scales’ correlations with criteria from 0.63 to 0.77 (Gulliksen 2016). Although the reliabilities are unsuitable for individual assessment, they are adequate for large-sample research, and such brief scales often exhibit enhanced validity, even given the upper limits of their validity coefficients.

### 5.2. Study 3 Results

#### 5.2.1. Study 3 Descriptive Statistics

The participants in Study 3 were similar to those in Study 1, where 92% were White, with 335 identified as female, 31% as male, and 3 as non-reported. Study 3’s sample had a larger proportion of freshmen and sophomores than either previous study at 71% versus 60% for Study 1 and 36% for Study 2. This sample had somewhat more psychology majors than Studies 1 and 2 at 33% versus 21% and 6.5%, respectively. Most of the measures’ descriptive statistics appeared stable across studies (shown in the third set of columns in Table 1).

#### 5.2.2. Study 3 Tests of Hypotheses

Did higher personal intelligence correlate with higher levels of positive choice regarding one’s major? The bottom rows of Table 2 show the Study 3 correlations between the TOPI and the CMS. The TOPI and CMS overall Positive Choice score correlated as *r* = 0.35, *p* < 0.001, similar to Study 2. The TOPI correlated positively with Confidence and Intrinsic Motivation scales (both *r*s = 0.28, *p*s < 0.001) and negatively with the Difficulty and especially Decision Avoidance (*r*s = −0.33 and −0.42, *p*s < 0.001).

Did the TOPI and the Choice of Major Scale correlate with better academic outcomes? As in Studies 1 and 2, TOPI scores correlated with higher GPA (Table 3, right). And students with higher TOPI scores considered a larger number of majors at the start of college, *r* = 0.13, *p* < 0.01, took less time to declare their major, *r* = −0.10, *p* < 0.05, and reported fewer thoughts of withdrawing from college, *r* = −0.12, *p* < 0.05, a pattern similar but not identical to that in Study 2. High PI students also exhibited less class absenteeism. The TOPI similarly correlated with the added “Preplanning for Advising” criterion at *r* = 0.37 and Match to Interests indices, *r* = 0.15, *p*s < 0.001 and 0.01. The CMS exhibited an *r* = 0.23, *p* < 0.001 with the Match to Interest index.

Did the academic criteria relate to the Big Six personality traits? Study 3 also allowed for the examination of the degree to which the HEXACO was related to the academic criteria (see Table 5). The TOPI correlated with Conscientiousness and Openness, *r* = 0.19 and 0.17, *p*s < 0.001, as it often does, but it did not correlate with Agreeableness, which it often has in the past. The TOPI correlated unexpectedly highly with Honesty at *r* = 0.43. The CMS Total scale similarly correlated with Conscientiousness, Openness, and Honesty.

The Big Six also exhibited meaningful relations with academic criteria: Honesty was related to higher GPA and to fewer absences from class, *r*s = 0.24 and −0.18, *p*s < 0.01. Extraversion and Openness to the number of majors considered, *r*s = 0.12 and 15, *p*s < 0.01. Conscientiousness was related to shorter time to declare a major, *r* = −0.10, *p* < 0.05; fewer absences from classes in the major *r* = −0.17 *p* < 0.01; higher GPA and better Match to Interest; and more preparation for advising, *r*s = 0.23, 0.16, 0.23 *p*s < 0.001, among other relations.

#### 5.2.3. Academic Advising Experience and Academic Adaptability

The TOPI and the CMS results also correlated with the Academic Advising Experience scale, which assessed students’ satisfaction or dissatisfaction with their advising experiences. The scores in Table 6 reflect the dissatisfaction–satisfaction of advising, school burnout, and academic adaptability. Students with higher scores on the TOPI and the CMS experienced less dissatisfaction with the advising process, less burnout, and more adaptability—especially with regards to learning. Neither the SAT or ACT exhibited such relationships.

#### 5.2.4. Incremental Validity of the Ability Measures

How did personal intelligence compare to SAT, a proxy measure of general intelligence, and the CMS? As noted earlier, the SAT correlates with general intelligence, *g*, about *r* = 0.73 to 0.82 (e.g., Coyle 2015; Frey 2019; Frey and Detterman 2004). We took advantage of the relation by recalculating the correlations for the TOPI, while partialing the influence of the SAT (as a proxy for *g*), to indicate whether the correlations with the TOPI remained statistically significant.

The TOPI continued to exhibit significant relations with the criteria even with the statistical control for the SAT. For example, the TOPI continued to correlate with the number of majors initially considered and (negatively) with thoughts about withdrawing, *r*s = 0.12 and −0.10, *p*s < 0.05 with SAT as a control. The TOPI still correlated with matching one’s personality to one’s major and fewer absences from classes in one’s major, *r*s = 0.14 and −0.11, *p* < 0.01 and < 0.05. Its strong relation with advising preplanning remained significant at *r* = 0.36, *p* < 0.001. The TOPI also correlated with overall GPA and GPA in the major over and above the SAT, *r*s = 0.13 and 0.23, *p*s < 0.01 and 0.001.

The TOPI partial correlations, controlling for the CMS, also remained significant in several instances. Controlling for the CMS, the TOPI correlated with alternative majors initially considered, *r* = 0.13, *p* < 0.05; grades overall and in the major, *r* = 0.12 and 0.21 *p*s < 0.01 and 0.001; and preplanning for advising, *r* = 30, *p* < 0.001. The same question asked using a regression approach yielded similar findings, and it is reported in the Technical Supplement (Mayer and Sylaska 2024, Section 4.1).

Was personal intelligence related to outcomes beyond the Big Six? To find out if the TOPI incrementally correlated above and beyond the Bix Six personality traits in Study 3, we examined the relative explanatory powers of the six HEXACO variables and the TOPI in hierarchical multiple regressions for each of the academic criterion items. After accounting for the effects of the Big Six in each model’s first step, entering personal intelligence in the second step significantly contributed to explained variance for (a) thinking of more alternatives to their current major (*R^2^_change_* = 0.01; *F*(1, 484) = 5.66, *p* = 0.018), (b) taking less time to declare their major (*R^2^_change_* = 0.01; *F*(1, 476) = 4.32, *p* = 0.038), (c) earning a higher overall GPA (*R^2^_change_* = 0.01; *F*(1, 483) = 7.02, *p* = 0.008), (d) earning a higher GPA in their major (*R^2^_change_* = 0.05; *F*(1, 482) = 25.08, *p* < 0.001), and (e) more preplanning for advising meetings (*R^2^_change_* = 0.08; *F*(1, 484) = 45.04, *p* < 0.001). Each of the Big Six (with the exception of Agreeableness) explained a significant proportion of variance for different criteria; four criteria, however, were related to neither the HEXACO nor the TOPI: the number of majors initially considered, hours spent studying for major courses or non-major courses, and seeking supplemental advising assistance. Details are provided in the Technical Supplement (Mayer and Sylaska 2024, Section 4.1).

Altogether, the results suggested that the TOPI often contributed incremental variance to relations, above and beyond the CMS, SAT, and HEXACO, depending upon the outcome measure involved.

## 6. Mini Meta-Analysis

We also conducted a mini meta-analysis (Goh et al. 2016) of the three studies to determine the overall relations of the TOPI and CMS with the academic lifespace indices. The estimated mean correlations across studies can be seen in the right-most columns of Table 4. We used a fixed-effects model to calculate an estimated *r* across studies by converting the three *r*s for a given relation to Fisher *z*s, taking a weighted average using the overall *n* of the study, and then converting the *z* back to an overall *r.* The *p* level was calculated using the overall *N* = 1262. Further details are in the Technical Supplement (Mayer and Sylaska 2024, Section 4.2).

Some highlights in Table 4 include that, across the three studies, the TOPI correlated with overall GPA and GPA in major, *r*s = 0.20 and 0.23, *p* < 0.001; fewer hours studying to attain those higher GPAs, *r*s = −0.06 and −0.11, *p*s < 0.05 and 0.001; number of majors considered initially, *r* = 0.10, *p* < 0.001; (shorter) time to declare major *r* = −0.07, *p* < 0.05; (fewer) changes in major and thoughts of withdrawing, both at *r* = −0.08, *p* < 0.01; and fewer absent classes in one’s major *r* = −0.07, *p* < 0.05.

The CMS similarly exhibited significant correlations for overall GPA and GPA in major, *r*s = 0.23 and 0.24 *p* < 0.001, but *more* rather than fewer hours to attain those higher GPAs in the major, *r* = 0.07 (for the for the non-major, *r* = −0.04, n.s.). More specific to the choice of major, the CMS correlated with fewer current thoughts of number of majors, *r* = −0.08, *p* < 0.01, and fewer thoughts of changing the major and of withdrawing, at *r* = −0.19 and −0.14, *p*s < 0.001.

## 7. General Discussion

The methods students employ to choose their college major and the extent to which their choice suits them is a matter of interest to the students themselves, psychologists, academic advisors, and those responsible for college policies. The initial work reported here developed the Choice of Major Scale to capture students’ phenomenology around choosing a major. Factor models on the combined Study 1 and 2 sample of *N* = 770 led to the development of an overall 26-item scale of Positive Major Choice that divided into six factors, with Confidence and Intrinsic motivation on the positive side and Difficulty and Decision Avoidance on the negative side (see Figure 1 for the full model). The model replicated well on the Study 3 sample of *N* = 492.

Students volunteering for Study 1 were higher in ability level and engagement in decision making than in Studies 2 and 3, resulting in a restriction of range in that sample on the TOPI (22% less variance) and, less dramatically, for the CMS. The findings of Study 1 were often consistent with those of Studies 2 and 3, but the range restriction appeared to attenuate the level and significance of the findings. For that reason, we emphasize results from Studies 2 and 3 and the average *r* across studies from the mini meta-analysis (Goh et al. 2016).

The key outcomes were the Academic Major Criterion Items used across the three studies that collectively indexed a student’s passage through deciding on their major(s). These lifespace-type items (a.k.a. biodata items) are externally verifiable in principle. Predictors of such outcomes typically exhibit meaningful correlations in the *r* = |0.05| to |0.25| range individually (Furnham 2017; Karas and West 1999; Mayer et al. 2024; Mount et al. 2000).

Our findings indicated the presence of a reasoning process described by personal intelligence on the TOPI and manifest in a student’s willingness to engage in thinking about what major is best for them in the CMS. In Studies 2 and 3, The TOPI and CMS overall correlated with one another in the *r* = 0.30 range, indicating that students’ personal intelligence co-occurred with their willingness to engage in reasoning about the major and the quality of their experience around the major they ultimately chose. Students with lower TOPI scores reported that their majors were more difficult on the CMS in all three studies, and they avoided decisions about their major in Studies 2 and 3.

Students who both possessed the ability to reason well about themselves (TOPI) and exhibited a willingness to apply such reasoning to their context (CMS) exhibited a reasonably consistent pattern of relations to the academic criteria across studies: They considered more alternative majors to start with, took less time declare their majors, changed their majors less frequently, and considered withdrawing from college less. They performed better in courses for their major and overall, exhibited fewer absences from classes, exhibited a closer match between their personal interests and their majors, and preplanned for meetings with their advisors by, for example, examining course options for upcoming semesters. More generally, in Study 3 (where the measures were taken), they were better protected against school burnout and were more satisfied with their advising experiences and appeared more adaptable to the college experience.

We also examined the six personality traits assessed by the HEXACO scales in Study 3. Although those were also correlated with some of foregoing criteria, regression analyses indicated that PI and CMS often added incremental variance over such standard measures.

### 7.1. Strengths and Limitations

Some findings concerning the TOPI and CMS and their relations to the Academic Major Criterion Items (ACMI) were inconsistent across the studies to a degree, suggesting that the individual studies, despite their substantial sample sizes, were individually underpowered given the levels of relationships obtained in some instances. In addition, the sample in Study 1 exhibited a restriction of range on the TOPI. To compensate for this, we also estimated the average correlation across studies in a mini meta-analysis. In addition, some newer questions concerning advising were present only in Study 3. Further development of the Academic Major Criteria Items may be helpful, as well as further replication. The ACMI consists of lifespace items—observable and potentially verifiable—and these have unique and valuable measurement properties. Although the correlation between lifespace GPA and GPA on transcripts are very high at *r* = 0.85 (Sticca et al. 2017), we note that they are not perfect, and this might have affected the results. Some readers may also wonder whether the correlations between PI and the criteria here would hold if emotional intelligence were controlled for. Although the two people-centered intelligences are conceptually distinct, their substantial empirical correlation suggests that emotional intelligence might well exhibit similar patterns. The distinction between the two likely needs a more focused set of empirical studies to draw out.

### 7.2. Implications for Educational Policy

Students who choose a suitable major in college are less likely to drop out, more likely to experience higher levels of well-being while in school, and perform better academically. Some research suggests that students who declared a major sooner in their college careers have higher rates of persistence and are more likely to graduate on time (e.g., Allen and Robbins 2010; Leppel et al. 2001). Such findings led the University System of Georgia, for example, to mandate that all entering students declare a major or meta-major (i.e., an area/cluster of majors) prior to beginning classes (Kafka 2019).

The original research used to justify such mandates, however, did not include clear comparisons of undeclared students with their declared counterparts (e.g., Graunke et al. 2006; Spight 2020). The present studies clarify that students who declare earlier may possess higher levels of personal intelligence and engage in more active consideration of their interests, as indicated on the Test of Personal Intelligence and the Choice of Major Scale. It is not clear whether mandates alone, without support to enhance students’ self-understanding, are sufficient to bring about improved performance because one can declare a major with or without having made a thoughtful or well-reasoned choice. Rather, the findings here suggest that good academic advising to help a student identify their interests and motivations may work well in tandem with any such requirements.

## 8. Conclusions

A student’s choice of major is determined by multiple factors, in part by parents, peers, cultural and economic issues, as well as by the quality of the department and professors who offer the major. The individual student also makes choices based on their interests, capabilities, and motivations. The present work examined these personality-related indicators in relation to students’ choice of college major. The capacity to reason about one’s personality—personal intelligence—contributed to good choices, as did the students’ engagement in an active decision-making process around the major, as measured by the Choice of Major Scale. Such findings provide evidence of how personal intelligence, as well as a willingness to reason about personality, contribute to people’s day-to-day lives. In the college setting, students who are lower in personal intelligence and who are decision-avoidant on the CMS may benefit from advisors and administrators who can help them better understand their interests and motives and assist them in matching those to their choice of major.

## Figures and Tables

**Table 1:** Confirmatory factor model fits to the three individual domain scales and overall Choice of Major Scale.

Models	*N*	No. of Factors	Items	Free Params	χ^2^	df	RMSEA	CFI	TLI	SRMR	r*_stnd_*
	CFA Fits of the Model to the Combined Liberal Arts and MTurk Samples, *N* = 770										
Individual Domains											
Satisfaction with Major	770	2	8	33	58.59	19	0.052	0.992	0.988	0.023	−0.65
Motivation in Major	770	2	9	38	73.486	25	0.050	0.988	0.983	0.033	0.09
Thinking about Major	770	2	9	37	61.78	26	0.042	0.989	0.985	0.027	−0.52
Overall—Hierarchical											
As-is	770	7	26	110	1475.58	293	0.072	0.931	0.923	0.066	−0.69 to 0.71
3 Double-Loaders	770	7	26	113	1170.91	290	0.063	0.949	0.942	0.056	−0.80 to 0.92
	CFA Cross-Validation on the PSYC Sample of Study 3, *N* = 492										
Overall—Hierarchical											
As-is	492	7	26	109	1117.33	293	0.076	0.931	0.923	0.074	−0.96 to 0.83
3 Double-Loaders	492	7	26	112	951.40	290	0.068	0.945	0.938	0.065	−0.81 to 0.90

Note. RMSEA—Root mean square error of approximation; CFI—Comparative Fit Index; TLI—Tucker–Lewis Index; SRMR—standardized root mean square residual; *r*_stnd_—estimated correlations among factors for the standardized model.

**Table 2:** Descriptive statistics for measures common to the three studies.

		Study 1 COLA Sample		Study 2 MTURK Sample		Study 3 PSYC Sample	
	Min–Max Possible	Mean	SD	Mean	SD	Mean	SD
**Indices of Ability**							
TOPI_MINI_MEAN	0.00 to 1.00	0.84	0.15	0.78	0.23	0.74	0.26
TOPI 4G-45	0 to 45	--	--	--	--	32.84	8.69
SAT (T-scored)	--	--	--	--	--	50.00	10.00
ACT (T-scored)	--	--	--	--	--	50.00	10.00
GPA	0.00 to 4.33	3.32	0.63	3.35	0.48	3.11	0.54
**Choice of Major Scale**							
Total (Positive Choice)	1.00 to 4.00	3.05	0.28	3.04	0.34	2.97	0.30
Satisfaction	1.00 to 4.00	3.41	0.40	3.36	0.48	3.21	0.44
Difficulty	1.00 to 4.00	1.98	0.43	2.10	0.64	2.14	0.51
Intrinsic Motivation	1.00 to 4.00	3.34	0.42	3.20	0.48	3.14	0.45
External Rewards	1.00 to 4.00	2.42	0.52	2.83	0.53	2.64	0.49
Decision Avoidance	1.00 to 4.00	1.70	0.45	1.81	0.58	1.84	0.49
Social Alignment	1.00 to 4.00	3.13	0.42	3.07	0.43	3.05	0.40
**Academic Major Criterion Items**							
**Decision Making**							
Majors Considered	0 to 78 ^a^	2.37	2.86	2.50	2.49	2.78	2.42
Majors Curr. Thinking of	0 to 78 ^a^	0.48	1.29	0.12	0.69	0.92	0.73
Time to Declare Major	0 to 9 sems.	1.36	1.74	2.23	2.11	1.50	1.90
Changes in Major (Number of)	0 to 4+ times	0.50	0.84	0.68	0.93	0.80	0.91
Thinking of Withdrawing	0 to 2 (N/S/Y)	0.18	0.45	0.33	0.60	0.56	0.75
**Grades**							
GPA Overall	0.00 to 4.33	3.32	0.63	3.34	0.49	3.11	0.54
GPA in Major	0.00 to 4.33	3.38	0.63	3.38	0.56	3.16	0.60
**Hours of Study**							
Hours of Study, Major	0.5 h to >10	4.29	2.30	5.20	3.03	4.68	2.45
Hours of Study, Non-Major	0.5 h to >10	3.08	2.06	3.20	2.54	3.27	2.10
**Enrollment and Absences**							
Extra Courses in Major	0 to 1 (N/Y)	0.80	0.40	0.62	0.49	0.70	0.46
Classes Absent in Major	1 to >15	2.28	2.15	4.20	4.92	3.19	2.87
Classes Absent, Non-major	1 to >15	3.44	3.03	4.65	4.94	4.20	3.25
**Control/Other**							
No. of Courses, Overall	1 to 41	14.41	9.61	19.28	12.48	11.02	6.98
No. of Courses, Major	1 to 21	6.19	4.79	10.03	6.16	6.04	4.53

^a^ Students selected from a list of 78 options; relative to the potential range, the obtained ranges for “Majors Considered” were 0–17 for Study 1 and 0–16 for Studies 2 and 3; for “Majors Currently Thinking of”, they were 0–11 for Study 1, 0–8 for Study 2, and 0–4 for Study 3.

**Table 3:** Correlations between the TOPI and Choice of Major Scale and its subscale across the three studies (*N* = 304, 466, and 492).

	TOPI ^1^	Choice of Major Scale						
		Total	Confid.	Difficult	Intrins	Ext. Rew	Avoid	Align
**Study 1**								
TOPI MINI	1.00							
Total CMS Score	0.03	1.00						
Confidence	0.01	0.78 ***	1.00					
Difficulty	−0.23 ***	−0.48 ***	−0.43 ***	1.00				
Intrinsic Motivation	0.09	0.81 ***	0.67 ***	−0.37 ***	1.00			
External Reward	−0.10	0.48 ***	0.09	0.07	0.13 *	1.00		
Decision Avoid.	−0.05	−0.72 ***	−0.64 ***	0.35 ***	−0.62 ***	−0.18 ***	1.00	
Social Alignment	−0.03	0.73 ***	0.48 ***	−0.28 ***	0.56 ***	0.20 ***	−0.43 ***	1.00
**Study 2**								
TOPI MINI	1.00							
Total CMS Score	0.33 ***	1.00						
Confidence	0.31 ***	0.80 ***	1.00					
Difficulty	−0.32 ***	−0.60 ***	−0.46 ***	1.00				
Intrinsic Motivation	0.21 ***	0.83 ***	0.63 ***	−0.42 ***	1.00			
External Reward	0.03	0.39 ***	0.18 ***	0.10 *	0.13 **	1.00		
Decision Avoid.	−0.46 ***	−0.68 ***	−0.52 ***	0.59 ***	−0.51 ***	−0.07	1.00	
Social Alignment	0.02	0.71 ***	0.54 ***	−0.25 ***	0.62 ***	0.21 ***	−0.30 ***	1.00
**Study 3**								
TOPI 4G-45	1.00							
Total CMS Score	0.35 ***	1.00						
Confidence	0.28 ***	0.80 ***	1.00					
Difficulty	−0.33 ***	−0.55 ***	−0.44 ***	1.00				
Intrinsic Motivation	0.28 ***	0.86 ***	0.69 ***	−0.39 ***	1.00			
External Reward	−0.01	0.40 ***	0.09	0.11 *	0.19 ***	1.00		
Decision Avoid.	−0.42 ***	−0.71 ***	−0.58 ***	0.53 ***	−0.57 ***	−0.13 **	1.00	
Social Alignment	0.18 ***	0.73 ***	0.49 ***	−0.24 ***	0.65 ***	0.22 ***	−0.40 ***	1.00

^1^ The TOPI MINI was used in Studies 1 and 2, and the TOPI 4 select longform was used in Study 3. * *p* < 0.05; ** *p* < 0.01, *** *p* < 0.001.

**Table 4:** Correlations with key criteria across studies.

		Study 1			Study 2			Study 3					Cross-Study Avg ^a^	
Area of Measure	Specific Measure	TOPI MINI	CMS Total	GPA	TOPI MINI	CMS Total	GPA	TOPI 4G-45	CMS Total	SAT	ACT	GPA	TOPI	CMS
**Indices of Ability**														
	Test of Personal Intelligence	1.00 **	0.03	0.21 **	1.00 **	0.33	0.19 **	1.00 **	0.35 **	0.12	0.10	0.20 **	1.00	0.26 ***
	Overall GPA	0.21 **	0.16 **	1.00 **	0.19 **	0.23 **	1.00 **	0.20 **	0.27 **	0.24 ***	0.27 **	1.00 **	0.20 ***	0.23 ***
**Academic Major Criterion Items (AMCI)**														
**Decision Making**	Majors Considered	0.01	0.04	0.11 *	0.12 *	0.03	0.03	0.13 **	0.02	−0.01	0.06	−0.07	0.10 ***	0.03
	Majors Currently Thinking of	−0.02	−0.17 *	−0.06	−0.06	−0.03	−0.02	0.00	−0.06	0.03	−0.02	−0.02	−0.03	−0.08 **
	Time to Declare Major	−0.04	−0.09	−0.06	−0.07	−0.08	−0.10 *	−0.10 *	−0.16 **	−0.22 **	−0.18 **	−0.12 **	−0.07*	−0.11 ***
	Changes in Major	−0.07	−0.09	−0.07	−0.16 **	−0.05	−0.14 **	0.00	−0.38 **	−0.09	0.00	−0.07	−0.08 **	−0.19 ***
	Thinking of Withdrawing	−0.02	−0.27 *	−0.13 *	−0.08	−0.03	−0.10 *	−0.12 *	−0.17 **	−0.18	−0.06	−0.18 **	−0.08 **	−0.14 ***
**Grades**	Overall GPA	0.21 **	0.16 **	1.00 **	0.19 **	0.23 **	1.00 **	0.20 **	0.27 **	0.24 ***	0.27 **	1.00 **	0.20 ***	0.23 ***
	Major GPA	0.18 **	0.16 **	0.64 **	0.19 **	0.23 **	0.72 **	0.29 **	0.30 **	0.24 ***	0.34 **	0.70 **	0.22 ***	0.24 ***
**Hours of Study**	Hours of Study, Major	−0.11	0.04	0.12 *	−0.01	0.20	0.10 *	−0.07	−0.03	0.01	−0.02	0.01	−0.06 *	0.07 *
	Hours of Study, Non-Major	−0.07	−0.02	0.07	−0.19 **	−0.01	0.09	−0.05	−0.09	−0.04	−0.14 *	0.03	−0.11 ***	−0.04
**Enrollments and Absences**	Extra Courses in Major	0.04	0.15 *	0.05	−0.05	0.01	0.00	0.04	0.10 *	0.03	0.07	0.10 *	0.01	0.08 *
	Classes Absent, Major	−0.09	−0.04	−0.32 **	−0.01	0.07	0.02	−0.12 **	−0.20 **	−0.06	0.03	−0.28 **	−0.07 *	−0.06 *
	Classes Absent, Non-Major	−0.04	−0.02	−0.22 **	−0.03	0.07	−0.02	−0.08	−0.13 **	−0.11	0.02	−0.27 **	−0.05	−0.03
**Advising**	Preplanning for Meetings	--	--	--	--	--	--	0.37 **	0.32 **	0.06	−0.04	−0.19 **	--	--
	Supplemental Assistance	--	--	--	--	--	--	0.02	0.13 *	−0.17	−0.01	−0.03	--	--
**Matching**	Match to Interest	--	--	--	--	--	--	0.15 **	0.23 **	0.01	0.08	0.08	--	--
**Control**	No. of Courses, Overall	0.00	0.09	0.07	0.24 **	0.11 *	0.10 *	0.08	0.09 *	0.07	0.10 *	0.09 *	0.12 ***	0.10 ***
	No. of Courses, Major	−0.02	0.13 *	0.12 *	0.12 **	0.02	0.07	0.00	0.06	0.02	0.03	0.09 *	0.04	0.06

GPA appears in the table twice, under “Indicies of Ability” and the “Academic Major Criterion Items” for the reader’s convenience in making comparisons. ^a^ The cross-study average was calculated as a mini meta-analysis using a fixed-effects model by converting the individual *r*s to Fisher *z*s, taking the average, weighted by overall study *N*, and then converting back to an *r* (e.g., Goh et al. 2016). * *p* < 0.05; ** *p* < 0.01; *** *p* < 0.001.

**Table 5:** Relations of the Big Six to criteria.

Key Criteria	Honesty	Emotionality	Extraversion	Agreeableness	Conscientiousness	Openness
**Indices of Ability and Choice**						
Test of Personal Intelligence	0.43 ***	0.11 *	0.19 ***	0.03	0.19 ***	0.17 ***
TOPI MINI	0.41 ***	0.06	0.15 **	0.03	0.20 ***	0.17 ***
SAT	0.12	−0.05	0.15 *	−0.03	0.15 *	0.10
ACT	−0.06	−0.09	−0.06	−0.09	0.01	0.19 ***
Choice of Major	0.29 ***	−0.07	0.28 ***	0.04	0.31 *	0.17 *
**Academic Major Criterion Items**						
**Decision Making**						
Majors Considered	0.05	0.04	0.12 **	−0.04	−0.10	0.15 **
Majors Curr. Thinking of	0.02	0.11 *	0.00	−0.03	−0.10	−0.03
Time to Declare Major	−0.01	0.03	0.03	0.00	−0.10 *	−0.03
Match to Interest	0.09	−0.01	−0.10 *	0.02	0.16 **	0.20 ***
Changes in Major	0.02	−0.03	−0.07	0.08	−0.13 *	0.01
Thinking of Withdrawing	−0.11 *	−0.02	−0.13 **	0.03	−0.13 **	−0.06
**Grades**						
Overall GPA	0.24 ***	0.05	0.13 **	0.05	0.23 ***	−0.08
Major GPA	0.24 ***	0.04	0.17 ***	0.02	0.14 **	0.01
**Hours of Study**						
Hours of Study, Major	0.05	−0.06	−0.03	0.04	0.09*	0.00
Hours of Study, Non-Major	−0.03	0.06	−0.05	0.10 *	−0.02	−0.03
**Enrollments and Absences**						
Extra Courses in Major	0.09 *	0.11 *	0.07	0.03	0.00	0.00
Classes Absent, Major	−0.18 ***	−0.03	−0.03	−0.06	−0.17 ***	0.07
Classes Absent, Non-Major	−0.21 ***	−0.03	0.07	−0.04	−0.22 ***	0.01
**Advising**						
Preplanning for Meetings	0.27 ***	0.04	0.21 ***	0.02	0.23 ***	0.09 *
Supplemental Assistance	0.11 *	−0.01	0.13 **	0.10 *	0.01	0.00
**Control**						
No. of Courses, Overall	0.08	−0.05	0.05	−0.03	0.13 **	0.06
No. of Courses, Major	0.08	−0.04	0.00	−0.03	0.12 **	0.03

* *p* < 0.05; ** *p* < 0.01; *** *p* < 0.001.

**Table 6:** Correlations of cognitive measures with (negative) advising experiences and adaptability.

	TOPI	CMS	GPA	SAT	ACT
Negative Advising Exp.	−0.26 ***	−0.32 ***	−0.07	−0.10	0.03
School Burnout	−0.16 ***	−0.36 ***	−0.29 ***	−0.12	−0.01
Adaptability—Overall	0.16 ***	0.39 ***	0.09 *	0.11	0.06
Adapt.—Learning	0.19 ***	0.45 ***	0.14 **	0.09	−0.01
Adapt.—Uncertainty	0.07	0.19 ***	0.01	0.10	0.10

* *p* < 0.05; ** *p* < 0.01; *** *p* < 0.001.

## Data Availability

A deidentified version of the key data presented in these studies will be available upon request.
